# Comparative Analysis of TAVR (Transcatheter Aortic Valve Replacement) and Surgical Valve Replacement for Low-Risk Patients

**DOI:** 10.7759/cureus.47234

**Published:** 2023-10-17

**Authors:** Usha Topalkatti, Ram Chandra Prasad, Bhagya Raju Koppu, Kalva Suchitra Reddy, Siddhanth Kumar Mekala, Rajarahulnaik Banothu, Hemanth Vasireddy, Renuka Sri Sai Peddireddi

**Affiliations:** 1 Internal Medicine, Spartan Health Sciences University, Vieux Fort, LCA; 2 Internal Medicine, MediCiti Institute of Medical Sciences, Hyderabad, IND; 3 Pediatric Medicine, Apollo DRDO (Defence Research and Development Organisation) Hospitals, Hyderabad, IND; 4 Internal Medicine, SVS (Sri Venkata Sai) Medical College and Hospital, Hyderabad, IND; 5 Internal Medicine, Indira Gandhi Medical College and Research Institute, Puducherry, IND

**Keywords:** aortic stenosis (as), tavr, surgical aortic valve replacement (savr), heart failure surgery, cardiothoracic & vascular surgery

## Abstract

“Aortic stenosis” (AS) refers to a cardiac condition in which the aortic valve narrows, creating an obstruction that hinders the flow of blood from the left ventricle to the aorta. This contraction of the arteries influences normal blood circulation, leading to elevated pressure within the left ventricle and potentially culminating in heart failure. The management of AS typically involves two primary treatments, i.e. “surgical aortic valve replacement” (SAVR) and “transcatheter aortic valve replacement” (TAVR). In both cases, the goal is to replace a dysfunctional aortic valve with a functional substitute. Presently, TAVR has gained much preference over SAVR in clinical practice. However, there is a dearth of comprehensive research directly comparing the real-world outcomes of TAVR and SAVR. In recent years, TAVR has emerged as an attractive alternative to SAVR, yet studies that provide a detailed comparison of their real-world solutions are limited. This review article assesses the mortality of patients who underwent TAVR vis-a-vis patients who underwent SAVR.

## Introduction and background

Aortic stenosis (AS) remains one of the major health concerns globally, with an increased prevalence due to the global aging population [1-3]. The prognosis of AS is unfavorable when symptoms manifest, as indicated by a five-year survival rate ranging from 15% to 50% [4]. Classification of risk categories (low, intermediate, high) for aortic valve replacement clinical practice is based on score levels specifically designed to assess surgical risks by organizations such as the Society of Thoracic Surgeons (STS), EuroSCORE, and EuroSCORE II [5]. Data extracted from the “Society of Thoracic Surgeons” (STS) registry reveals that 93.9% of patients who underwent surgical aortic valve replacement (SAVR) belonged to the low- to intermediate-risk groups [6]. However, current understanding suggests that patients diagnosed with AS who possess a high to intermediate surgical risk experience more advantages from the use of “transcatheter aortic valve replacement” (TAVR) in comparison to only receiving medicinal intervention [7-10]. Our present understanding suggests that TAVR and SAVR exhibit similar rates of mortality and stroke in patients having a low surgical risk [11]. These low-risk patients experience below-average rates of rehospitalization [10]. TAVR has been widely demonstrated to be comparable with SAVR [12-13], even better than SAVR among prohibitive and high-risk patients [14]. Consequently, due to its remarkable success rates, TAVR has emerged as a preferred choice for aortic valve replacement [14]. An important question is to find the best way to treat low-risk patients with serious AS, who make up about 40% of the patients who seek treatment. Recent studies recommend that patients aged 65 and older who are suffering from severe AS symptoms should consider TAVR [6,15].

TAVR has become an effective treatment option for patients with severe AS who are at high to moderate risk for SAVR [16]. While deciding which treatment choices are best, it is important to take into account not only the high cost of TAVR but also how well the treatment works overall [17]. However, in clinical practice, the current procedures still tend to favor SAVR when patients have a reduced risk of experiencing negative outcomes [18]. The main objective of this narrative review is to analyze randomized controlled trials (RCTs) that compare the outcomes of transcatheter aortic valve replacement (TAVR) and surgical aortic valve replacement (SAVR). This research attempts to evaluate the safety, effectiveness, and overall success of a specific treatment in patients categorized as having a low to intermediate risk for surgery [19-20].

## Review

TAVR and SAVR in chronic kidney disease (CKD) patients

Virtanen et al. [21] conducted a comparative study examining the success rate of TAVR and SAVR in patients suffering from moderate to severe chronic kidney disease (CKD). All participants included in the study satisfied the predetermined criteria for either treatment option [21]. The study examined patients who were below the age of 80 and had Society of Thoracic Surgery scores (STS scores) ranging from 4 to 8. This score signifies that they were in an intermediate-risk group, with the presence of symptomatic severe aortic stenosis (AS). Virtanen et al. used data obtained from the "German Aortic Valve Registry" in their analysis, using the propensity score approach to establish a matched sample [16,21]. The study included a total of 374 patients who underwent SAVR and 704 patients who underwent TAVR. The aim was to observe the relative probability of survival among patients with TAVR compared to SAVR, with emphasis on one-year survival outcomes. Additionally, Virtanen et al. [21] also examined a range of clinical implications, including the need for dialysis, the need for pacemaker insertion, the incidence of vascular problems, the probability of myocardial infarction, and the tendency for bleeding events. Significantly, there was no noticeable difference in the age distribution among the survivors in both cohorts.

Upon observing one-year survival rates, the researchers found no significant difference in the survival rates of the patients. While there were marginal distinctions in short-term and one-year survival rates, these disparities did not attain statistical significance, with a short-term survival rate of 96.4% and a one-year survival rate of 86.2% [21]. The outcomes illuminated that TAVR exhibited a heightened predisposition for necessitating pacemaker insertion and vascular complications. TAVR-related vascular complications encompass injuries, access site issues, stenosis, thrombosis, embolization risks, and rare but severe outcomes like aortic dissection, influencing the blood vessels during transcatheter aortic valve replacement.

Conversely, SAVR was associated with an increased risk of myocardial infarction (MI), bleeding events requiring blood transfusions, prolonged stays within the intensive care unit (ICU), and overall hospital admissions. Patients who underwent SAVR also exhibited a greater likelihood of requiring temporary hemolysis management, although the demand for long-term hemolysis management remained infrequent and exhibited similarity between both treatment groups [21].

TAVR and SAVR in low-risk patients

Khan et al. conducted an assessment of the comparative efficacy of TAVR and SAVR within a cohort of low-risk patients. This examination drew upon data from seven reputable academic sources [22], and it predominantly centered on evaluating mortality rates at the 30-day and 12-month intervals, without delving into the specific underlying causes of mortality. The study encompassed a substantial dataset comprising a total of 4,859 patients.

Strikingly, TAVR demonstrated a significant 40.1% reduction in the 30-day mortality risk associated with various causes when contrasted with SAVR [22]. This difference was substantiated through rigorous statistical analysis, which yielded a notable distinction between the two treatment groups. The relative risk (RR) confidence interval, established at 95% accuracy, ranged from 0.38 to 0.92, and the calculated p-value amounted to 0.02. However, it is essential to note that the study did not identify a statistically significant variance in the overall mortality within one year between the two cohorts. The subset of patients who opted for TAVR experienced a noteworthy reduction in mortality, measuring at 21%, signifying that their risk of mortality was 0.79 times lower in comparison to those who did not undergo the procedure. The corresponding confidence interval, signifying the level of statistical certainty, spanned from 0.57 to 1.09 at a 95% confidence level. The associated p-value, indicative of statistical significance, was 0.15.

The findings further illuminate that patients who elected to undergo TAVR exhibited a remarkable 36% lower likelihood of encountering a stroke within the ensuing 30 days when compared to those who did not opt for the surgical intervention. This discrepancy in outcomes was notably more favorable for the TAVR group. Moreover, individuals who chose TAVR over SAVR demonstrated a significantly diminished risk of experiencing moderate to severe acute kidney injury (AKI), with a striking 56% reduction. Nonetheless, it is worth highlighting that among those who underwent TAVR, there was an elevated incidence of vascular complications necessitating permanent pacemaker (PPM) placement. Khan et al. [22] also conducted an examination to ascertain whether patients who underwent TAVR were at a heightened risk of developing endocarditis. The findings indicate that only a small number of endocarditis cases were detected, and no conspicuous disparities in statistical significance were discerned between the 30-day and one-year time frames.

Randomized controlled trials (RCTs) meta-analysis

Kolte et al. conducted a comparative analysis of the TAVR procedure in contrast to the SAVR technique, with a focal point on patients characterized by a low-risk profile and a diagnosis of severe aortic stenosis (AS) [23]. The researchers embarked on an exhaustive exploration of electronic repositories to identify relevant randomized controlled trials (RCTs), successfully identifying four clinical studies that met their stringent eligibility criteria, collectively involving a cohort of 2,887 patients. The allocation of patients to either TAVR or SAVR was carried out through a randomization process. The demographic characteristics of the subjects revealed a mean age of 75.4 years, accompanied by an average Society of Thoracic Surgeons Predicted Risk of Mortality (STS-PROM) score of 2.3%. This STS-PROM score signifies a moderate degree of surgical risk in the patient population.

Remarkably, the analysis also unveiled that TAVR was associated with a reduced risk of all-cause mortality (2.1% compared to 3.5%) as well as mortality specifically attributed to cardiac issues (1.6% versus 2.9%) within a one-year timeframe. Patients who underwent TAVR also exhibited a diminished likelihood of experiencing new-onset atrial fibrillation or exacerbation of pre-existing cases. Furthermore, they exhibited a reduced risk of encountering serious or life-threatening bleeding, as well as stage 2 or 3 acute kidney injury (AKI). However, it is noteworthy that the TAVR cohort displayed an increased likelihood of experiencing moderate to severe paravalvular leakage and necessitating permanent pacemaker implantation in comparison to their SAVR counterparts. This observation holds significant clinical relevance and merits careful consideration. Importantly, no substantial differences were detected between the two procedures concerning severe vascular complications, occurrences of endocarditis, or the necessity for aortic valve re-intervention. Based on the insights derived from this comprehensive meta-analysis, it becomes unequivocally apparent that the study furnishes compelling evidence in favor of the proposition that TAVR represents a superior alternative to SAVR for patients grappling with severe AS who meet the stipulated criteria for aortic valve replacement with a bioprosthetic valve. This assertion is particularly pronounced when scrutinizing the subgroup of patients categorized as low-risk individuals [24]. The findings collectively suggest that TAVR is associated with reduced rates of overall mortality and cardiovascular-related fatalities within a one-year timeframe, specifically among the subset of patients under investigation.

Meta-analysis comparing TAVR and SAVR in low-risk patients with severe aortic stenosis

Kolkailah et al. delved into the nuanced distinctions between SAVR and TAVR procedures within a cohort of patients diagnosed with AS and assessed as possessing a low surgical risk profile [25]. Aortic stenosis stands as a pivotal global health concern, and the selection between SAVR and TAVR in low-risk patient populations has been a persistent subject of discourse and investigation. To embark on this evaluative journey, an extensive and rigorous search was conducted across multiple databases to discern pertinent randomized controlled trials (RCTs). The comprehensive analysis encompassed participants extracted from five distinct clinical studies, further supplemented by data from an ongoing study, culminating in a cumulative participant count of 2,818 individuals. It is paramount to acknowledge the variance in the overall quality of the amassed medical evidence, spanning the spectrum from very high to very low.

The primary focus of scrutiny revolved around pivotal clinical outcomes, with an emphasis on total mortality rates, the incidence of strokes, and post-procedure readmissions to healthcare facilities within a 30-day timeframe. The culmination of this meticulous data analysis pointed towards minimal, if not negligible, disparities between TAVR and SAVR, particularly in the context of patients harboring low-risk profiles. These distinctions encompassed multifaceted dimensions such as all-cause mortality, stroke occurrence, and the prevalence of myocardial infarction (MI). Moreover, our extensive research efforts indicated a potential advantage for TAVR, manifesting as a reduced likelihood of hospital readmissions when juxtaposed against SAVR. However, it remains imperative to underscore the intrinsic nuances within these findings, encapsulated by the presence of confidence intervals that include the possibility of insignificance. This underscores the requisite caution demanded during the interpretation of these results.

The study also unveiled a noteworthy association between TAVR and a heightened probability of necessitating a permanent pacemaker implant. However, discerning the impact of TAVR on the duration of hospital stays remained beyond the scope of this investigation. On the flip side, TAVR emerged as the favorable choice in several facets when compared to SAVR. Current medical research lends substantial support to the assertion that TAVR is intricately linked to reduced incidences of hemorrhage, acute kidney injury (AKI), and atrial fibrillation, marking these as notable advantages of the TAVR approach.

TAVR versus SAVR in severe aortic stenosis with reduced left ventricular ejection fraction (LVEF)

Jalava et al. undertook a meticulous investigation, focusing on a specific cohort of patients afflicted with severe AS coupled with reduced left ventricular ejection fraction (LVEF), and delved into the comparative outcomes of valve replacement procedures, specifically TAVR and SAVR within this distinctive patient group [26]. Given the challenging prognosis associated with this medical condition, their paramount aim was to discern the potential alterations in patient survival rates contingent on the therapy administered. To accomplish this, the reviewers adeptly employed propensity score matching methodologies to analyze the essential attributes of patients exhibiting an LVEF of 50% or lower. The dataset encompassed a total of 6,463 patients. Within the surgical cohort, a noteworthy 20.8% (876 patients) exhibited a diminished LVEF, mirroring the TAVR cohort where 27.7% (452 patients) shared this characteristic. Particularly noteworthy was their revelation of a compelling correlation between reduced LVEF levels and diminished survival rates, underscoring the pivotal role of LVEF as a predictive factor for patient outcomes. This association held true even after an average follow-up duration of 3.6 years for each patient within the study. It is significant to highlight that the TAVR group displayed a reduced 30-day mortality rate (3.1% versus 7.9%) in comparison to the SAVR group, as evident in our analysis of 255 patient pairs with closely aligned propensity scores. This distinction persisted when comparing the overall mortality rates between the two groups, and it is underscored by the statistical significance of the associated p-value, which stood at 0.038. However, it is of paramount importance to note that survival rates after both one year and four years were identified as analogous for both treatment modalities. The calculated average survival time ratio between SAVR and TAVR stood at 1.002, signifying the absence of a significant difference in intermediate-term survival outcomes. This conclusion is robustly supported by a p-value of 0.964%. In light of these findings, it becomes evident that a decline in LVEF is intricately linked to an augmented susceptibility to illness and heightened mortality risk following TAVR or SAVR procedures. TAVR exhibits a potential advantage over SAVR when evaluating 30-day mortality rates, but in the intermediate term, the survival rates achieved by both therapeutic approaches manifest striking similarities.

Meta-analysis evaluating TAVR versus SAVR in patients at high risk

Nagaraja et al. conducted a meta-analysis to assess and compare the effectiveness of TAVR and SAVR in patients who were considered ineligible for SAVR [27]. Further, Zaleska-Kociecka et al. focused on significant outcomes such as the incidence of AKI [28], as well as the rates of strokes and heart attacks, mortality rates at 30 days and one year, and mortality rates within one month. The RCTs did not reveal any statistically significant differences between the cohort that underwent SAVR and the cohort that underwent TAVR. Other risk factors included mortality rates at 30 days and one year, as well as the occurrence of cardiovascular events like heart attacks and strokes. The outcomes derived from non-randomized controlled trials (non-RCTs) pointed out a relatively higher occurrence of aortic regurgitation in patients following TAVR, particularly after their hospital discharge [29]. However, research encompassing essential parameters such as the occurrence of myocardial infarction (MI), stroke, acute renal failure necessitating hemodialysis, 30-day mortality, and the requirement for a pacemaker revealed no notable distinctions between the two procedures [30-31].

A noteworthy observation pertained to the TAVR, which exhibited a diminished necessity for blood transfusions and a decreased occurrence of atrial fibrillation onset. This study utilized a combination of randomized and observational data to evaluate various outcome measures, including the 30-day mortality rate, one-year mortality rate, stroke rate, myocardial infarction rate, and AKI rate. The results underwent a rigorous adjustment technique to accurately consider the baseline characteristics of the trial participants. Consequently, the results of this investigation underscore TAVR as a promising and effective alternative for patients for whom SAVR is not deemed suitable, offering outcomes that align in various key aspects. It is worth noting that the research was conducted using an open-access approach, enabling the widespread dissemination and utilization of its findings.

## Conclusions

Examining diverse studies comparing TAVR and SAVR suggests varied outcomes across different patient profiles. In the context of patients with low to moderate surgical risk, TAVR exhibits similar effectiveness in terms of death rates, stroke prevention, and overall clinical outcomes. However, it is relevant to keep in mind that TAVR is associated with a higher probability of requiring pacemaker implantation and experiencing vascular problems. The review suggests that there is a comparable one-year survival rate between TAVR and SAVR for patients diagnosed with chronic kidney disease. However, significant differences exist in terms of complications, with TAVR being connected with vascular difficulties and SAVR being linked to MI and bleeding events. Significantly, TAVR exhibits a substantial decrease in the risk of death within 30 days, incidence of stroke, and occurrence of acute renal damage in patients with low risk, hence highlighting its beneficial advantages. Furthermore, the evaluation of individuals suffering from severe AS and a decrease in the ability of the left ventricle to expel blood indicates that TAVR may provide benefits in terms of 30-day mortality rates when compared to SAVR. However, the long-term survival rates of the two procedures seem to be similar. The meta-analysis conducted on high-risk patients who are not suitable for SAVR reveals that there are no statistically significant differences in outcomes between TAVR and SAVR. Both treatments exhibit distinct benefits and concerns. In general, this combination of findings highlights the increasing significance of TAVR as a potentially favorable option, especially in certain groups of patients. It also recognizes the need to make customized decisions based on the patient's risk profiles and medical conditions.

## References

[1] Goldbarg SH, Elmariah S, Miller MA, Fuster V (2007). Insights into degenerative aortic valve disease. J Am Coll Cardiol.

[2] Nkomo VT, Gardin JM, Skelton TN, Gottdiener JS, Scott CG, Enriquez-Sarano M (2006). Burden of valvular heart diseases: a population-based study. Lancet.

[3] Iung B, Baron G, Butchart EG (2003). A prospective survey of patients with valvular heart disease in Europe: The Euro Heart Survey on Valvular Heart Disease. Eur Heart J.

[4] Vahanian A, Alfieri O, Andreotti F (2012). Guidelines on the management of valvular heart disease (version 2012). Eur Heart J.

[5] Rogers T, Thourani VH, Waksman R (2018). Transcatheter aortic valve replacement in intermediate- and low-risk patients. J Am Heart Assoc.

[6] Nishimura RA, Otto CM, Bonow RO (2017). 2017 AHA/ACC Focused Update of the 2014 AHA/ACC Guideline for the Management of Patients With Valvular Heart Disease: A Report of the American College of Cardiology/American Heart Association Task Force on Clinical Practice Guidelines. Circulation.

[7] Cahill TJ, Terre JA, George I (2020). Over 15 years: the advancement of transcatheter aortic valve replacement. Ann Cardiothorac Surg.

[8] Mack MJ, Leon MB, Thourani VH (2019). Transcatheter aortic-valve replacement with a balloon-expandable valve in low-risk patients. N Engl J Med.

[9] Thyregod HG, Ihlemann N, Jørgensen TH (2019). Five-year clinical and echocardiographic outcomes from the NOTION randomized clinical trial in patients at lower surgical risk. Circulation.

[10] Popma JJ, Deeb GM, Yakubov SJ (2019). Transcatheter aortic-valve replacement with a self-expanding valve in low-risk patients. N Engl J Med.

[11] Braghiroli J, Kapoor K, Thielhelm TP, Ferreira T, Cohen MG (2020). Transcatheter aortic valve replacement in low risk patients: a review of PARTNER 3 and Evolut low risk trials. Cardiovasc Diagn Ther.

[12] Siemieniuk RA, Agoritsas T, Manja V (2016). Transcatheter versus surgical aortic valve replacement in patients with severe aortic stenosis at low and intermediate risk: systematic review and meta-analysis. BMJ.

[13] Smith CR, Leon MB, Mack MJ (2011). Transcatheter versus surgical aortic-valve replacement in high-risk patients. N Engl J Med.

[14] Fusari M, Bona V, Muratori M, Salvi L, Salis S, Tamborini G, Biglioli P (2012). Transcatheter vs. surgical aortic valve replacement: a retrospective analysis assessing clinical effectiveness and safety. J Cardiovasc Med (Hagerstown).

[15] Mesnier J, Panagides V, Nuche J, Rodés-Cabau J (2022). Evolving indications of transcatheter aortic valve replacement: where are we now, and where are we going. J Clin Med.

[16] Mas-Peiro S, Faerber G, Bon D (2022). Propensity matched comparison of TAVI and SAVR in intermediate-risk patients with severe aortic stenosis and moderate-to-severe chronic kidney disease: a subgroup analysis from the German Aortic Valve Registry. Clin Res Cardiol.

[17] Tsigkas G, Despotopoulos S, Makris A, Koniari I, Armylagos S, Davlouros P, Hahalis G (2018). Transcatheter versus surgical aortic valve replacement in severe, symptomatic aortic stenosis. J Geriatr Cardiol.

[18] Alsara O, Alsarah A, Laird-Fick H (2014). Advanced age and the clinical outcomes of transcatheter aortic valve implantation. J Geriatr Cardiol.

[19] Witberg G, Lador A, Yahav D, Kornowski R (2018). Transcatheter versus surgical aortic valve replacement in patients at low surgical risk: a meta-analysis of randomized trials and propensity score matched observational studies. Catheter Cardiovasc Interv.

[20] Gargiulo G, Sannino A, Capodanno D (2016). Transcatheter aortic valve implantation versus surgical aortic valve replacement: a systematic review and meta-analysis. Ann Intern Med.

[21] Virtanen MP, Eskola M, Jalava MP (2019). Comparison of outcomes after transcatheter aortic valve replacement vs surgical aortic valve replacement among patients with aortic stenosis at low operative risk. JAMA Netw Open.

[22] Khan MS, Mir T, Ullah W (2020). Comparing transcatheter aortic valve replacement (AVR) with surgical AVR in lower risk patients: a comprehensive meta-analysis and systematic review. Cardiol Res.

[23] Kolte D, Vlahakes GJ, Palacios IF, Sakhuja R, Passeri JJ, Inglessis I, Elmariah S (2019). Transcatheter versus surgical aortic valve replacement in low-risk patients. J Am Coll Cardiol.

[24] Petronio AS, Capranzano P, Barbato E, Piazza N, Baumbach A, Haude M, Windecker S (2016). Current status of transcatheter valve therapy in Europe: results from an EAPCI survey. EuroIntervention.

[25] Kolkailah AA, Doukky R, Pelletier MP, Volgman AS, Kaneko T, Nabhan AF (2019). Transcatheter aortic valve implantation versus surgical aortic valve replacement for severe aortic stenosis in people with low surgical risk. Cochrane Database Syst Rev.

[26] Jalava MP, Savontaus M, Ahvenvaara T (2022). Transcatheter and surgical aortic valve replacement in patients with left ventricular dysfunction. J Cardiothorac Surg.

[27] Nagaraja V, Raval J, Eslick GD, Ong AT (2014). Transcatheter versus surgical aortic valve replacement: a systematic review and meta-analysis of randomised and non-randomised trials. Open Heart.

[28] Zaleska-Kociecka M, Dabrowski M, Stepinska J (2019). Acute kidney injury after transcatheter aortic valve replacement in the elderly: outcomes and risk management. Clin Interv Aging.

[29] Soong EL, Jing OY, Ho JS (2021). Transcatheter aortic valve replacement for aortic regurgitation in Asians. AsiaIntervention.

[30] Shroff GR, Frederick PD, Herzog CA (2012). Renal failure and acute myocardial infarction: clinical characteristics in patients with advanced chronic kidney disease, on dialysis, and without chronic kidney disease. A collaborative project of the United States Renal Data System/National Institutes of Health and the National Registry of Myocardial Infarction. Am Heart J.

[31] Rahman MS, Sharma R, Brecker SJ (2015). Transcatheter aortic valve implantation in patients with pre-existing chronic kidney disease. Int J Cardiol Heart Vasc.

